# Constraint-based analysis of gene interactions using restricted boolean networks and time-series data

**DOI:** 10.1186/1753-6561-5-S2-S5

**Published:** 2011-05-28

**Authors:** Carlos HA Higa, Vitor HP Louzada, Tales P Andrade, Ronaldo F Hashimoto

**Affiliations:** 1 Institute of Mathematics and Statistics, University of Sao Paulo, Rua do Matao 1010, 05508-090, Sao Paulo - SP, Brazil; 2 School of Computing, Federal University of Mato Grosso do Sul, Cidade Universitaria, 79070-900, Campo Grande - MS, Brazil

## Abstract

**Background:**

A popular model for gene regulatory networks is the Boolean network model. In this paper, we propose an algorithm to perform an analysis of gene regulatory interactions using the Boolean network model and time-series data. Actually, the Boolean network is restricted in the sense that only a subset of all possible Boolean functions are considered. We explore some mathematical properties of the restricted Boolean networks in order to avoid the full search approach. The problem is modeled as a Constraint Satisfaction Problem (CSP) and CSP techniques are used to solve it.

**Results:**

We applied the proposed algorithm in two data sets. First, we used an artificial dataset obtained from a model for the budding yeast cell cycle. The second data set is derived from experiments performed using HeLa cells. The results show that some interactions can be fully or, at least, partially determined under the Boolean model considered.

**Conclusions:**

The algorithm proposed can be used as a first step for detection of gene/protein interactions. It is able to infer gene relationships from time-series data of gene expression, and this inference process can be aided by a *priori* knowledge available.

## Background

One of the goals of Systems Biology is to study the various cellular mechanisms and components. In many cases these mechanisms are complex, where some of the interactions between the proteins are still unknown. To represent these interactions it is common to use gene regulatory networks (GRN). There are several models of GRN, both discrete and continuous. The simplest discrete model was introduced by Kauffman [1] and its known as *Boolean network*. Later, this model was modified to express uncertainty giving rise to the *probabilistic Boolean network*[2,3]. Friedman introduced *Bayesian networks*[4] as a probabilistic tool for the identification of regulatory data and showed that they can reproduce certain known regulatory relationships. Among the continuous models we can cite the *ordinary differential equations* which was suggested several decades ago [5]. For a more detailed review about models of gene regulatory networks see [6].

Models of gene regulatory networks help us study biological phenomena (e.g. cell cycle) and diseases (e.g. cancer). Therefore, revealing such networks, or at least some of its connections, is an important problem to address. The ability to uncover the mechanisms of GRN has been possible due to developments in high-throughput technologies, allowing scientists to perform analysis on the DNA and RNA levels. The most common type of data provided by these technologies are gene expression data (microarray). The biological systems are notoriously complex. Determining how the pieces of this puzzle come together to create living systems is a hard challenge known as *reverse engineering*, which is the process of elucidating the structure of a system by reasoning backwards from observations of its behavior [7]. However, in many cases, GRN cannot be precisely unraveled due to measurement noise and the limited number of data sets compared to the number of genes that are involved.

The most common approach to reverse engineering GRN is to use gene expression data. For example, Marshall et al. [8] considered the approach of using time-series data and probabilistic Boolean networks (PBNs) as a model of GRN. In fact, there are many other works about inferring PBNs [9-13].Computational algebra approaches were also proposed in [14,15]. Jarrah et al. [16,17] used *polynomial dynamical systems* for reverse engineering of GRN. One good survey for inferring GRN from time-series data can be found in [18]. Some algorithms use additional information from heterogeneous data sources, e.g. genome sequence and protein-DNA interaction data, to assist the inference process. Hecker et al. [19] presents a good review of GRN inference and data integration.

Usually, an inference algorithm aims to construct one single network which is believed to be the true network. The issue is that the inverse problem is ill-posed, meaning that several networks could explain (or generate) the data set given as the input for the algorithm. In fact, a study for validation of GRN inference procedures can be found in [20]. The problem becomes more complicated if we take into account the noise that may be present in the data and the small amount of samples. For this reason, our approach aims to analyze several networks that could explain the data. By analyzing the similarities among these networks, we propose a confidence measure of the regulatory relationship between the genes.

In this paper, we present an algorithm for analysis of gene interactions. Although this analysis is directly connected to the process of inference of gene regulatory networks, the main goal of this work is not the inference. The idea is that the algorithm could be used as a first step of an inference process, that is, a pre-processing of the data, in order to support an inference process. To perform the analysis, the algorithm generates a limited number of *consistent* networks (to be explained in the next section). Unlike any inference algorithm, our algorithm does not take these networks as the final result (the true network). It uses these networks to perform the analysis of gene interactions.

The algorithm is based on Boolean networks and time-series gene expression. Actually, the Boolean networks are called *restricted* in the sense that not all Boolean functions are allowed in the model. Restricting the network reduces the search space, which can be significant, since the inverse problem is very complex. This restricted model allows us to find constraints that turn our problem into what can be seen as a Constraint Satisfaction Problem (CSP) and CSP techniques can be used to find feasible solutions, that is, networks. The time-series data allows us to observe part of the dynamics of the system. These observations are used to generate the constraints of the CSP.

A challenge always presented in any gene regulatory model is its usefulness. It would be interesting if a model could help biological experiments in understanding gene interactions. The model here presented is capable of inferring some of these connections from time-series data of gene expressions, and this inference process is aided by all a *priori* knowledge available. What we envisage with our method is a model that points out which connections should be inspected in the wet lab that would constrain as many other connections as possible and consequently could facilitate some biological experiments.

## Methods

### Restricted Boolean network model

A Boolean network (BN) is defined by a set **X** = {*x*_1_, *x*_2_,…,*x_n_*} of *n* Boolean variables and a set **F** = {*f*_1_, *f*_2_,…,*f_n_*} of *n* Boolean functions. In the case of GRN the variables are called genes. Each gene *x_i_*, *i* = 1,…,*n*, can assume only two possible values: 0 (OFF) or 1 (ON). The value of the gene *x_i_* at time *t* + 1 is determined by genes *x*_*j*_1_(*i*)_, *x*_*j*_2_(*i*)_,…,*x*_*j_k_i__*(*i*)_ at time *t* through a Boolean function *f_i_* : {0, 1}*^k_i_^* → {0,1}. Given that, there are *k_i_* genes assigned to gene *x_i_*, and the mapping *j_k_* : {1,…,*n*} → {1,…,*n*}, *k* = 1,…,*k_i_* determines the “wiring” of *x_i_*[21]. This way,

*x_i_*(*t* + 1) = *f_i_*(*x*_*j*_1_(*i*)_(*t*), *x*_*j*_2_(*i*)_(*t*),…,*x*_*j_k_i__*(*i*)_(*t*)) . (1)

We assume that all genes are updated synchronously by the functions in **F** assigned to them and this process is repeated. The artificial synchrony simplifies computation while preserving the qualitative, generic properties of global network dynamics [22,23]. A *state* of the network at time *t* is a binary vector *s*(*t*) = (*x*_1_(*t*),…,*x_n_*(*t*)). Therefore, the number of states is 2*^n^*, labeled by *s*_0_, *s*_1_,…,*s*_2^*n*^–1_. The dynamics of the network is represented by the transition between states. This model is deterministic given that there is a single Boolean function to regulate each gene. Because of the finite number of states and the deterministic behavior, some of the states may be visited cyclically. These states form what is known by the *attractor* of the BN. The states outside the attractor are called *transient* states. The transient states together with the corresponding attractor states form the *basin of attraction* of that attractor.

In the case of restricted Boolean networks, the regulatory relationships is represented by a matrix *A*_*n*×*n*_ using the following convention: *a_ij_* = 1 for a positive regulation on gene *x_i_* from gene *x_j_*; *a_ij_* = –1 for a negative regulation on *x_i_* from *x_j_*; For the remaining cases *a_ij_* = 0. The Boolean function *f_i_* is defined according to the matrix *A* and the values of the genes *x_j_*, *j* = 1,…,*n*, at time *t*:(2)

We call the summation ∑_*j*_*a_ij_x_j_*(*t*) the *input* of *x_i_* at time *t*. Besides the regulatory relationships of the matrix *A*, each gene can have a self-degradative behavior implying that its value is set to 0 whenever its input is null. Observe that not all Boolean functions can be represented using (2) and that is why the Boolean network is called “restricted”. It is also worth to notice here that each gene *x_i_* depends only on the *i*-th row of *A*.

### Constraint satisfaction problem

A constraint satisfaction problem (CSP) is defined by a set of variables *X* = {*x*_1_, *x*_2_,…,*x_n_*}; a collection of finite sets ***D*** = {*D*_1_, *D*_2_,…,*D_n_*}, where *D_i_* is the domain set for *x_i_*; and a collection of set of constraints ***C*** = {*C*_1_, *C*_2_,…,*C_m_*} restricting the values that all variables can assume simultaneously, where each set *C_i_* involves constraints of a subset of variables and specifies the allowable combinations of values for that subset. A solution of a CSP is an assignment, to every variable *x_i_*, a value from its domain *D_i_* such that all constraints in *C* are satisfied [24,25].

CSPs defined on finite domains are usually solved by search algorithms meaning systematically assigning possible values to variables and verifying whether all constraints may be satisfied or not. The most used techniques are variants of *backtracking*, *constraint propagation*, and *local search*[24-27]. In these search algorithms, the assignment process requires an order in which the variables are considered. In addition, after selecting a variable, it is necessary to decide the order in which a value is picked up from its domain (to assign to it). In fact, there are many heuristics for variable and value ordering [24,27]. If one is interesting in generating a sample of uniform solutions, a good heuristic for variable and value ordering may be, firstly, select randomly a variable (with uniform probability), and, then choose randomly (again, with uniform probability) a value from its domain. There are many CSP solvers in the literature; the one we are using is from Gecode project (http://www.gecode.org) [27].

### Analysis of gene interactions using CSP

One important remark about the Boolean model presented by Equation (2) is that each row in the regulation matrix *A* is independent from each other. Using this property, instead of applying the CSP to find all the possible solutions for the *A* matrix, we can apply the CSP to find all the feasible rows *r_i_* for each gene *x_i_*. In this way, the time complexity of the algorithm is reduced by decreasing the number of possible combinations from 3^*n*^2^^ (number of possible matrices) to *n* · 3*^n^* (total number of possible rows). Thus, in this context, the gene interaction problem considered in this paper can be modeled as a set of *n* CSPs. For each gene *x_i_* (*i* = 1,2, …,*n*), the problem *P_i_* is defined by the set of variables *R_i_* = {*a*_*i*,1_, *a*_*i*,2_,…,*a*_*i,n*_} (corresponding to the *n* entries of the *i*-th row of the regulation matrix); a collection of domain sets ***D****_i_* = {*D*_*i*,1_, *D*_*i*,2_,…,*D*_*i,n*_} (each *D*_*i,j*_ = {–1,0,1}); and a set of constraints obtained by considering all successions of states in the time-series data (see next subsection).

In order to analyze the gene interactions, since there may be a combinatorial explosion in generating the rows, we consider some of them (a random sampling process) to perform the analysis. In fact, as the number of genes grows, the problem becomes intractable and there could be too many consistent networks for a given data set. We would like to highlight that we are not concerned in generate all the consistent networks, but a limited number of them in order to perform an analysis of the gene interactions.

In situations where a large number of genes is considered and a small number of time-series data is available, one can reduce the number of genes to perform the gene interaction analysis by employing clustering analysis [28-31] or feature selection algorithms [32], and/or building small subnetworks by using the paradigm of growing seed genes [11,33]. Of course, one can still use prior knowledge by selecting a small number of genes involved in a specific biological process (e.g., cell cycle division, metabolic pathway).

### Constraints generation for the CSP

The algorithm was designed under the assumption that the gene expression data were generated by a biological system which can be modeled as a restricted Boolean network. Let **S** = {*S*(1), *S*(2),…,*S*(*m*)} be a set of *m* time-series gene expression profiles, where *S*(*t*) ∈ {0,1}*^n^* for *t* = 1,…,*m*. The algorithm aims to analyze networks that produce the sequence

*S*(1) → *S*(2) → ⋯ → *S*(*m*) . (3)

When the network produces the time-series data we say that the network is *consistent* with the data.

Naturally, there may exist several consistent networks for a single sequence. That is, the inverse problem is a “one-to-many” or ill-posed problem, and this is very difficult to handle.

One naïve way to solve this ill-posed problem is to find all possible networks by a full search algorithm. In fact, Lau et al. [34] proposed a “smart” full search algorithm to enumerate all possible networks. Here, in this paper, we explore some mathematical properties of the restricted Boolean networks in order to avoid this full search approach.

The algorithm aims to analyze the interactions between the genes through the information provided by the time-series sequence, which can be seen as a state transition sequence of the corresponding BN. These time-series data and the restricted Boolean network model are used to generate the constraints of the CSP, as we show next.

#### First constraints set

The first set of constraints is generated by analyzing the sample in triplets, *S*(*t* – 1), *S*(*t*) and *S*(*t* + 1). An important point to notice here is that if two consecutive states *S*(*t* – 1) and *S*(*t*) differ only in one single gene *x_k_*, then any gene *x_i_* that had its value changed from *S*(*t*) to *S*(*t* + 1) is directly regulated by *x_k_*. To illustrate this situation, consider the time-series data (Table 1). Looking at the time points *S*(1) and *S*(2) we observe that only *x*_2_ had its value changed (from 1 to 0). Now, looking at *S*(2) and *S*(3) we can see that *x*_3_ was turned to 1. Following the restricted Boolean network model, this change was caused, necessarily, by the gene *x*_2_. In fact, *x*_2_ inhibits *x*_3_ at time *t* = 1 and it is self degraded at time *t* = 2, allowing *x*_1_ to activate *x*_3_ at time *t* = 3. Using this approach, we state the following proposition.

**Table 1:** Time-series data

	*x*_1_(*t*)	*x*_2_(*t*)	*x*_3_(*t*)	*x*_4_(*t*)
*S*(1)	1	1	0	0

*S*(2)	1	0	0	0

*S*(3)	1	0	1	0

*S*(4)	1	0	1	1

*S*(5)	0	0	1	1

A small example of time-series data containing only four genes.

**Proposition 1.***Let S*(*t* – 1), *S*(*t*) *and S*(*t* + 1) *be three consecutive states according to the restricted Boolean network model. If S*(*t* – 1) *and S*(*t*) *differ by a single gene x_k_, then for each gene x_i_ such that x_i_*(*t*) ≠ *x_i_*(*t* + 1) *we have that x_k_ regulates x_i_ directly, that is, a_ik_* ≠ 0.

*Proof*. Suppose that *S*(*t* – 1) and *S*(*t*) differ by a single gene *x_k_*, and that there is at least one gene *x_i_* such that *x_i_*(*t*) ≠ *x_i_*(*t* + 1). As *x_i_*(*t*) ≠ *x_i_*(*t* + 1), the summations ∑_*j*_*a_ij_x_j_*(*t* – 1) and ∑_*j*_*a_ij_x_j_*(*t*) have different signs. Given that *x_k_* is the only gene possessing different values in *S*(*t* – 1) and *S*(*t*), this difference signal must have been caused by *x_k_*. Therefore, *a_ik_* ≠ 0.

#### Second constraints set

To generate the second set of constraints, the algorithm takes into account two consecutive states, *S*(*t*) and *S*(*t* + 1). There is one important observation here: only the active genes at time *t* can possibly regulate genes at time *t* + 1. This fact becomes clear when we look at Equation (2). The active genes can give us an insight of which genes are regulating other gene, although the type of the regulatory relationship can not be determined. However, the input given by the summation in Equation (2) can help us to determine the regulatory relationships. For example, if we observe that a gene *x_i_* changes its value from 0 (at time *t*) to 1 (at time *t* + 1), we can deduce that the input for gene *x_i_* is positive at time *t* and only the active genes at time *t* are responsible for this positive input. Following this logic, the algorithm generates all possible combinations of regulatory relationships using the active genes such that the input of gene *x_i_* at time *t* is coherent to the values of *x_i_* at time *t* + 1. More formally, we can state the following proposition.

**Proposition 2.***For each gene i, the state transition from x_i_*(*t*) *to x_i_*(*t* + 1) *generates constraints for variables a_ij_ according to* Table 2.

**Table 2:** Possible transitions

*x_i_*(*t*)	*x_i_*(*t* + 1)	constraint for *a_ij_*
1	0	

0	1	

0	0	

1	1	

All possible transitions from *x_i_*(*t*) to *x_i_*(*t* + 1) and their respective generating constraints for *a_ij_*.

*Proof*. Let us first prove the first constraint, that is, if *x_i_*(*t*) = 1 and *x_i_*(*t* + 1) =0, then . According to Equation (2), the only way to change the state of gene *x_i_* from 1 (at time *t*) to 0 (at time *t* + 1) is if its input . If we just consider the genes *x_j_* that are active at time *t*, that is, those which *x_j_*(*t*) = 1, we can rewrite this constraint as . Analogously, one can prove the other constraints given in Table 2.

To exemplify, consider the data in Table 1 where *t* = 3, that is, *S*(3). At this time, there are two active genes, *x*_1_ and *x*_3_. These genes are the only ones that can contribute to the sign of the input for each gene for the next time. If we look at gene *x*_4_, we observe that its value turned from 0 to 1. According to Equation (2), the input must be positive in this case, that is, . Considering only the active genes at time *t* = 3, we must have *a*_41_ + a_43_ > 0. Therefore, neither *a*_41_ or a_43_ can take the value –1, only 1 or 0 (not both). The same procedure can be applied to all genes and then, the constraints for the CSP are generated.

#### Third constraints set

This set is generated by analyzing any two pairs of consecutive states in the time-series data. Let *t*_1_ and *t*_2_ be two time points in the time-series data:

⋯*S*(*t*_1_) → *S*(*t*_1_ + 1) → ⋯ → *S*(*t*_2_) → *S*(*t*_2_ + 1) ⋯.

Now, let us suppose that *S*(*t*_1_) and *S*(*t*_2_) are very similar. Hence, the difference between *S*(*t*_1_ + 1) and *S*(*t*_2_ + 1) must be caused by the differentially expressed genes of their predecessors. For instance, let us suppose that *S*(*t*_1_) and *S*(*t*_2_) differ in one single gene:(4)

And the succession occurs as stated:(5)

Therefore, the difference between *S*(*t*_1_ + 1) and *S*(*t*_2_ + 1) in this case must be caused by the change on *x*_4_. In this step, the algorithm checks how each gene changed in the two pairs of consecutive states.

In our example, let us concentrate on gene *x*_1_. It was inhibited in the first pair and had no change in the second pair. Let *I* be the total input generated by those genes with similar expression in *S*(*t*_1_) and *S*(*t*_2_), *M* be the input generated by *x*_4_ in *S*(*t*_1_), and  be the input generated by *x*_4_ in *S*(*t*_2_). Therefore, to explain the changes of *x*_1_ in the two pairs, we must have:(6)

If *a_ij_* represents the influence of gene *x_j_* over *x_i_*, we can calculate I, *M* and  as follows:(7)

*M* = *a*_14_ · 0 = 0 and (8)(9)

Henceforth,(10)

This result implies that the entry *a*_14_ of the matrix must have value 1.

If *S*(*t*_1_) and *S*(*t*_2_) differ in more than one gene, we can still generate hypotheses of regulation. In fact, this step tries to construct a system of inequalities with the inputs of each gene for every combination of two consecutive pairs. More formally, we can state the following proposition.

**Proposition 3.***Let t_a_ and t_b_ be two different time instants. The state transitions from x_i_*(*t_a_*) *to x_i_*(*t_a_* + 1) *and from x_i_*(*t_b_*) *to x_i_*(*t_b_* + 1) *generate constraints for variables a_ij_ according to* Table 3.

**Table 3:** Possible transitions

*x_i_*(*t_a_*) → *t_a_* + 1	*x_i_*(*t_b_*) → *x_i_*(*t_b_* + 1)	Constraints for *a_ij_*
1→0	0→1	

0→1	0→0	

1→1	0→0	

0→0	0→1	

0→1	1→0	

1→1	1→0	

0→0	1→1	

1→0	1→1	

All possible transitions for *x_i_*(*t_a_*) → *t_a_* + 1 and *x_i_*(*t_b_*) → *x_i_*(*t_b_* + 1) and their respective generating constraints for *a_i__j_*.

*Proof*. Let us first prove the first constraint, that is, if *x_i_*(*t_a_*) = 1, *x_i_*(*t_a_* + 1) = 0, *x_i_*(*t_b_*) = 0, and *x_i_*(*t_b_* + 1) = 1, then

Considering the state transition from *x_i_*(*t_a_*) = 1 to *x_i_*(*t_a_* + 1) =0, by Proposition 2, we have that . From set theory, we can write the index set of all active genes at time *t_a_*, *A*(*t_a_*) = {*j* : *x_j_*(*t_a_*) = 1}, as a union of two disjoints sets:

Hence,

Analogously, from the transition from *x_i_*(*t_b_*) = 0 to *x_i_*(*t_b_*) = 1, we have that

Now subtracting the last two inequalities, we have

The other constraints can be obtained in a similar way.

### Bar chart of connection frequencies

For a fixed row *r_i_*, the algorithm generates a collection of consistent rows *R_i_* = {*r*_*i*1_, *r*_*i*2_,…,*r_im_*} from the constraints generated by the time-series data and the CSP solver. Each consistent row  has *n* entries , each one corresponding to a one type of connection (inhibition, no connection or activation) on gene *x_i_* from gene *x_j_*. Thus, we can estimate the frequency of all possible connections for the entry *a_ij_* of the regulation matrix by computing the frequency of entries –1, 0 and 1 of all , for *k* = 1,2,…,*m*.

We can exhibit the frequency of different types of connections on gene *x_i_* from gene *x_j_*, by showing the estimated frequencies of –1, 0 and 1 for *a_ij_* using a bar chart, as we will see in the next section. Evidently, for a fixed row *r_i_*, *determined connections* on gene *x_i_* will appear with frequency 100% in all rows *R_i_*; while *partially determined connections* on gene *x_i_* will have, at least, one type of connection (inhibition, no connection or activation) with frequency 0%; and, for *undetermined connections*, all relationships will have nonzero frequencies.

### Interactions rank

One way to validate our results is to use this bar chart. To do so, we rank the interactions found by the connection frequencies and compare the most relevant ones to known interactions found in the literature. By searching through the literature, the direction of some interactions could not be determined. For instance, in some cases we know that there is an interaction between two genes *x_i_* and *x_j_*, but we do not know whether *x_i_* is activating/inhibiting *x_j_* or vice-versa. Therefore, we rank undirected gene interactions by adding the frequency of different types of connections in both ways. For example, the interaction of genes *x_i_* and *x_j_* is ranked according to the equation:(11)

where *inh*(*x_i_* ← *x_j_*) and *act*(*x_i_* ← *x_j_*) denote the estimated frequency of –1 (inhibition) and 1 (activation), respectively, for the entry *a_ij_* obtained from the set of the rows in *R_i_*.

For a set of *n* genes, we rank *n*^2^/2 gene interactions. Typically, the interactions with the highest rank are used in order to search for interactions already known in the literature.

### Inducing a connection

An interesting application of the bar chart of connection frequencies would be the answer of the following question: which *non-determined* (partially determined or undetermined) connection, once determined, would constrain as many other connections as possible? In an experimental context, a method that can point which connection would aggregate more “knowledge” to the network if determined leads to an empirical construction of optimal GRNs.

To investigate this point, a simple exhaustive search was implemented in the space of consistent rows *R_i_* for a gene *x_i_*, according to the following steps:

1. Set a *non-determined* (partially determined or undetermined) connection *a_ij_* of the set *R_i_* as a determined connection with a value *v* of its domain;

2. Set ;

3. Construct the bar chart connection frequencies of ;

4. Compute *score*(*a_ij_*,*v*) = *score*(*x_i_*) = ∑*_j_ determination*(*x_i_*,*x_j_*) on the constrained set , where *determination*(*x_i_*, *x_j_*) is 0 if the connection *x_i_* ← *x_j_* is undetermined, 0.5 if it is partially determined, and 1 if it is completely determined;

5. Repeat Steps 1-4 for all non-determined connections and all their domain values. In the limit, 3*^g^* will be tested, where *g* is the number of non-determined connections;

6. Chose *a_ij_* = *v* that has the highest *score*.

By the end of these steps, we can say that connection *a_ij_* = *v* determines as many other connections as possible for gene *x_i_*.

## Results and discussion

### Budding yeast cell cycle model

**Figure 1:**
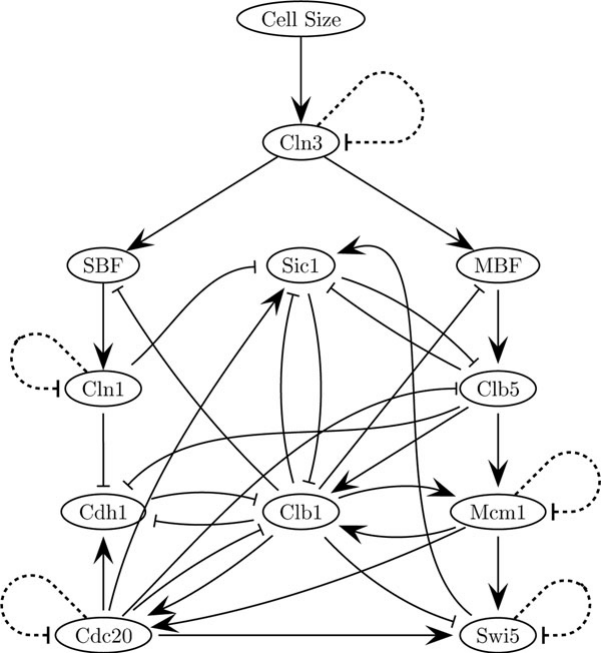
**Yeast network** The cell cycle network of the budding yeast.

We applied the algorithm in an artificial data set extracted from a model for the budding yeast (*Saccharomyces cerevisiae*) cell cycle. The model, proposed by Li et al. [35], is based on a network of eleven regulators as shown in Figure 1. The eleven genes *x*_1_,…,*x*_11_ are *Cln3*, *MBF*, *SBF*, *Cln1*, *Cdh1*, *Swi5*, *Cdc20*, *Clb5*, *Sic1*, *Clb1*, and *Mcm1*, respectively. The “cell-size” node was introduced just to indicate a checkpoint to start the cell-cycle process.

Considering the restricted Boolean network model, Li et al. [35] studied the dynamics of the network. They found that there are seven attractors, shown in Table 4. In this table, each row represents an attractor where the first column indicates the size of the basin of attraction. There is one big basin composed by 1,764 (or ≈ 86% of) states. According to Li et al. [35], the corresponding attractor is the biological G_1_ stationary state.

**Table 4:** Attractors

Basin size	Cln3	MBF	SBF	Cln1	Cdh1	Swi5	Cdc20	Clb5	Sic1	Clb1	Mcm1
1,764	0	0	0	0	1	0	0	0	1	0	0
151	0	0	1	1	0	0	0	0	0	0	0
109	0	1	0	0	1	0	0	0	1	0	0
9	0	0	0	0	0	0	0	0	1	0	0
7	0	1	0	0	0	0	0	0	1	0	0
7	0	0	0	0	0	0	0	0	0	0	0
1	0	0	0	0	1	0	0	0	0	0	0

The seven attractors of the budding yeast cell-cycle network.

Biologically, the cell-cycle sequence starts when the cell commits to division by activating Cln3. To simulate the cell cycle, they started the process by “exciting” the G_1_ stationary state with the cell size signal, that is, inducing the gene Cln3 to an active state. Applying Equation (2) to simulate the process it was observed that the system goes back to the G_1_ stationary state. The temporal evolution of the states, presented in Table 5, follows the cell-cycle sequence, going from excited G_1_ state (Start) to the S phase, the G_2_ phase, the M phase, and finally to the stationary G_1_ state. This is the biological trajectory or pathway of the cell-cycle network. The states presented in Table 5 are used as the artificial time-series data to perform the analysis using our algorithm.

**Table 5:** Temporal evolution

Time	Cln3	MBF	SBF	Cln1	Cdh1	Swi5	Cdc20	Clb5	Sic1	Clb1	Mcm1	Phase
1	1	0	0	0	1	0	0	0	1	0	0	Start
2	0	1	1	0	1	0	0	0	1	0	0	G_1_
3	0	1	1	1	1	0	0	0	1	0	0	G_1_
4	0	1	1	1	0	0	0	0	0	0	0	G_1_
5	0	1	1	1	0	0	0	1	0	0	0	S
6	0	1	1	1	0	0	0	1	0	1	1	G_2_
7	0	0	0	1	0	0	1	1	0	1	1	M
8	0	0	0	0	0	1	1	0	0	1	1	M
9	0	0	0	0	0	1	1	0	1	1	1	M
10	0	0	0	0	0	1	1	0	1	0	1	M
11	0	0	0	0	1	1	1	0	1	0	0	M
12	0	0	0	0	1	1	0	0	1	0	0	G_1_
13	0	0	0	0	1	0	0	0	1	0	0	Stationary G_1_

Temporal evolution of states for the budding yeast cell-cycle network.

#### Results for the budding yeast artificial data

**Figure 2:**
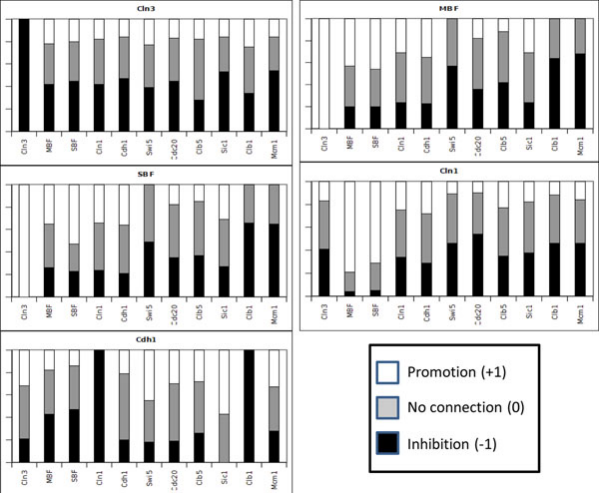
**Connection frequencies for the Budding Yeast data - 1** Frequency of the relationships in the consistent networks for the Budding Yeast artificial data. The frequencies of connections to each gene from all other genes were created by the application of the described algorithm and by a random determination of one connection. The determined connections are exhibited by only one color (black, white or gray), and the partially determined connections exhibited by two colors. We have generated 100 rows for each gene for the frequency connection analysis. The results for the remaining genes are shown in Figure 3.

**Figure 3:**
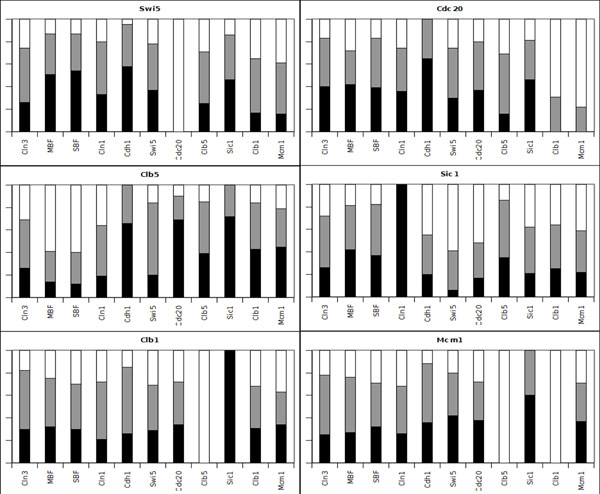
**Connection frequencies for the Budding Yeast artificial data - 2** Results for the genes *Swi5*, *Cdc20*, *Clb5*, *Sic1*, *Clb1* and *Mcm1*.

**Figure 4:**
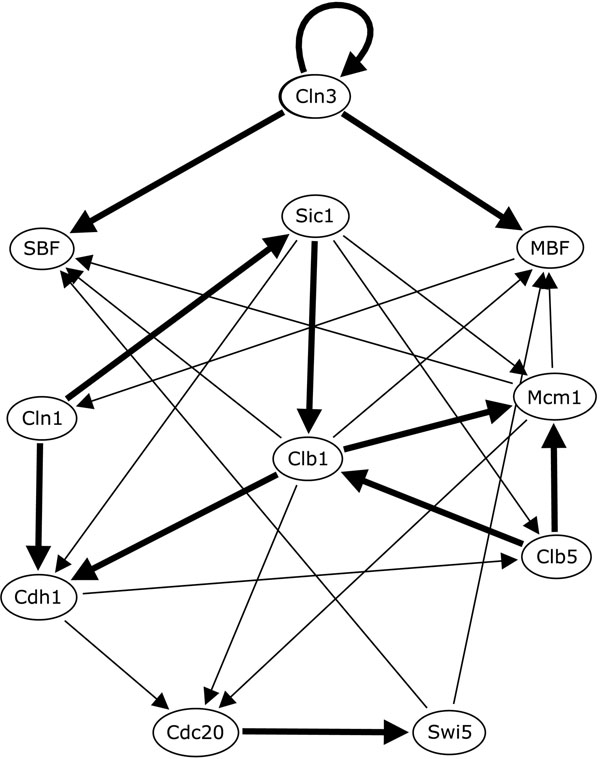
**Determined and partially determined connections** The determined (bold arrows) and partially determined connections (light solid arrows) inferred by the consecutive application of the algorithm in the budding yeast artificial data (the arrows do not necessarily indicate activation).

For each gene *x_i_*, the algorithm generates a collection of consistent rows *R_i_* using the time-series data (the 13 states presented in Table 5) to generate the constraints of the CSP. If we compute the frequency of the types of connections, we are able to assigning probabilities of connection for each pair of genes. In Figures 2 and 3 we show the frequency of different types of connections to each gene *x_i_* from all other genes. From these figures, we can see that the algorithm was capable of identifying 11 determined connections and 13 partially determined connections. The results are shown in Figure 4. Note that, in this figure, the arrows do not necessarily indicate activation.

Using these frequencies, we rank the undirected gene interactions using Equation 11. As an example, the 10 highest ranks are present in Table 6.

**Table 6:** Rank table

Genes interacting	Rank
Clb5 and Mcm1	1.72
Cln3 and SBF	1.70
Clb5 and Clb1	1.68
Cln3 and MBF	1.66
CLN1 and Sic1	1.66
Sic1 and Clb1	1.66
Swi5 and Cdc20	1.65
Clb1 and Mcm1	1.61
Cdh1 and Clb1	1.55
CLN1 and Cdh1	1.54

The 10 highest gene interactions ranks found by the application of the algorithm on the yeast cell cycle network.

**Figure 5:**
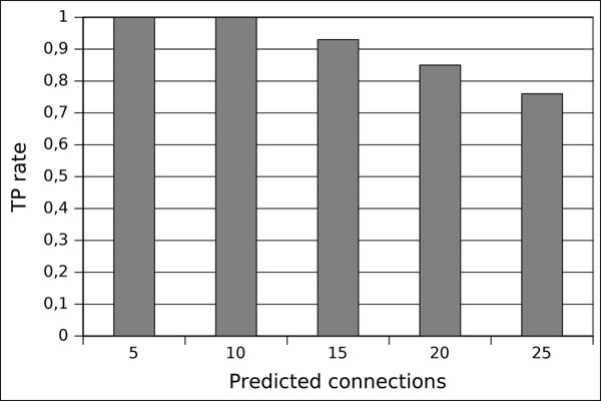
**True positive (yeast)** Validation of the proposed algorithm on the yeast cell cycle network. Plot of the true positive rate of predicted gene interactions computed from the highest ranks.

To validate our results, we consider a variable number of highest gene interactions ranks, from 5 to 25, and verify how many of these are present in the yeast cell cycle network (Figure 1), which allow us to compute a true positive rate, shown in Figure 5. This figure shows a true positive rate between 75% and 100% for different quantities of predicted gene interactions.

### HeLa cells

The immortal HeLa cell line is one of the oldest and most widely distributed human cell line. These cells are derived from cervical cancer cells of an African-American woman named Henrietta Lacks, who died in 1951. We applied our algorithm in a data set provided by Whitfield et al. [36] where the gene expression during the human cell cycle was characterized using cDNA microarrays. We used one of the five experiments consisting of 48 samples representing approximately three cycles and selected 20 well-characterized cell cycle genes. According to [36], each gene is assigned to a cell cycle phase shown in Table 7. The expression profiles of the 20 genes presented a cyclical pattern through the three cell cycles. Since our algorithm deals with Boolean values, we had to discretize the gene expression. To this end, we simply computed, for each gene *x_i_*, the mean *m_i_* of the expression profile. Then, for the gene *x_i_*, all the values exceeding the value of *m_i_* were set to 1, and the remaining values were set to 0. After this operation, each gene preserved its cyclical pattern, now in the Boolean domain.

**Table 7:** HeLa genes

Phase	Genes
G1/S	*CCNE1*, *E2F1*, *CDC6* and *PCNA*
S	*RFC4*, *DHFR*, *RRM2* and *RAD51*
G2	*CDC2*, *TOP2A*, *CCNF* and *CCNA2*
G2/M	*STK15*, *BUB1*, *CCNB1* and *PLK1*
M/G1	*PTTG1*, *RAD21*, *VEGFC* and *CDKN3*

The 20 well-characterized cell cycle genes and the respective phase where their expression peaks.

Another pre-processing step was to split the data into three data sets, one for each cycle. Considering the sample **S** = {*S*(1), *S*(2),…,*S*(48)}, we identified the binary state vectors that represent the attractors of the system. For example, the sequence of states *S*(1),…,*S*(5) are very similar and we consider them as equal states and represent them as a one singleton attractor (an attractor composed by a single state). The same approach was taken regarding the sequences of states *S*(16), *S*(17), *S*(18); *S*(30), *S*(31), *S*(32) and *S*(43),…,*S*(48). Although the binary states in the sequence may be not exactly the same, we are assuming that this difference is caused by the noise in the data. These singleton attractors are similar to the G_1_ stationary state of the budding yeast cell cycle model [35].

We identified three cell cycles *C*_1_ = *S*(6),…,*S*(17); *C*_2_ = *S*(19),…,*S*(31) and *C*_3_ = *S*(33),…,*S*(47). Therefore, to apply the algorithm on this data, we considered the three cell cycles separately. To analyze our results, for a fixed gene *x_i_*, we considered the union of all sets of rows *R_i_*, obtained from the application of the algorithm to each cell cycle, and then compute the connection frequencies.

#### Results for the HeLa cells

Comparing the three cycles present in the time-series data, we can see some effects of noise in the gene expression measurements. Supposedly, the three cycles should be equal, but there are minor differences among them. According to [36], the cells utilized in the microarray experiment, by the time of *C*_3_, could not be in the same cycle phase, compromising the experiment. Therefore, we did not utilize the data from *C*_3_ in our analysis.

The work of Whitfield et al. [36] makes also possible to add some biological knowledge to our algorithm. If we consider that the genes of the initial phases do not interact with genes of later phases, we reduce the set of possible rows. We can add this biological knowledge by setting some values of each row *r_i_* as 0 according to the information in Table 8. This way, we generate two sets of data. One without biological knowledge (named here as *ρ*_0,*i*_) and another with biological knowledge (*ρ*_1,*i*_).

**Table 8:** Gene set interactions

Genes in G1/S* are not regulated by genes in G2/M
Genes in G1/S are not regulated by genes in M
Genes in G2/M are not regulated by genes in G1/S
Genes in G2/M are not regulated by genes in S
Genes in M* are not regulated by genes in G1/S

Gene set interactions that are set as code 0 in *ρ*_1,*i*_. Set G1/S* does not contain gene CCNE1 and set M* does not contain gene CDKN3, since these two genes seem to interact with each other.

**Figure 6:**
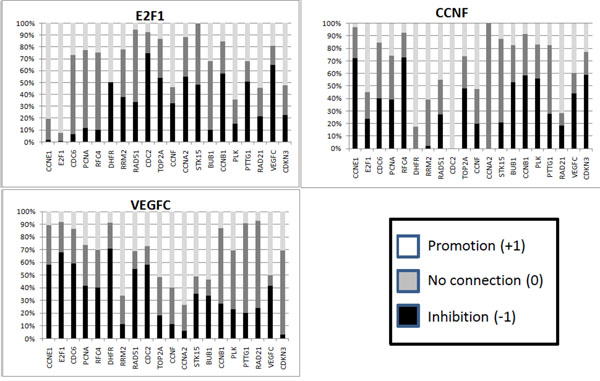
**Connection frequencies for the HeLa cells data - 1** Bar chart of connection frequencies of 3 genes from the HeLa cells data, using *ρ*_0,*i*_.

**Figure 7:**
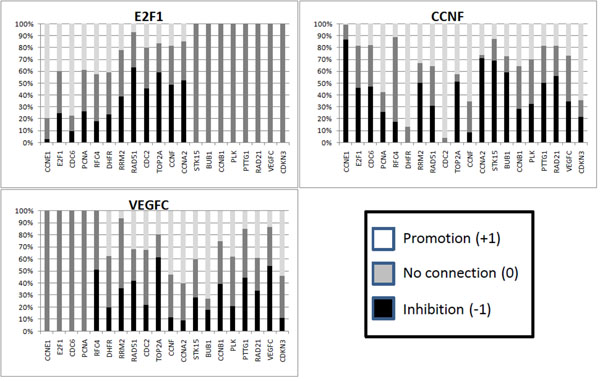
**Connection frequencies for the HeLa cells data - 2** Bar chart of connection frequencies of 3 genes from the HeLa cells data, using *ρ*_1,*i*_.

The algorithm is independently executed using the cell cycles *C*_1_ and *C*_2_ as the input data, generating a set of 10,000 rows for each gene *x_i_*. We take the union of the two set of rows obtained from both cell cycles and plotted a bar chart to observe the connection frequencies (partially shown in Figures 6 and 7, other charts are present in Additional file 1 and Additional file 2).

From the frequency analysis of all rows *r_i_*, (*i* = 1,2,…,*n*), we rank the undirected interactions. Tables 9 and 10 show the 10 highest gene interactions ranks. To validate these interactions we sought information about them through the literature. The tables also show the reference, when the information was found.

**Table 9:** Rank table

Genes interacting	Rank	Reference
DHFR and TOP2A	1.85	
DHFR and RAD21	1.8	
CCNE1 and CDC2	1.78	[37,38]
CCNE1 and CCNF	1.75	
RAD51 and STK15	1.75	
BUB1 and PTTG1	1.75	
RRM2 and CDC2	1.74	[39]
CDC2 and STK15	1.74	
E2F1 and DHFR	1.71	[40,41]
CCNE1 and RAD51	1.69	

The 10 highest gene interactions ranks found by the application of the algorithm on the HeLa cells data, considering *ρ*_0,*i*_.

**Table 10:** Rank table

Genes interacting	Rank	Reference
RRM2 and TOP2A	2	
RRM2 and CDC2	1.82	
CDC2 and CCNF	1.78	[42]
CCNF and CCNA2	1.72	
CCNE1 and CDC2	1.68	[37,38]
CDC2 and RAD21	1.68	
CDC2 and PTTG1	1.68	[43]
TOP2A and CCNF	1.67	
DHFR and TOP2A	1.66	
PCNA and RFC4	1.66	[44,45]

The 10 highest gene interactions ranks found by the application of the algorithm on the HeLa cells data, considering *ρ*_1,*i*_.

**Figure 8:**
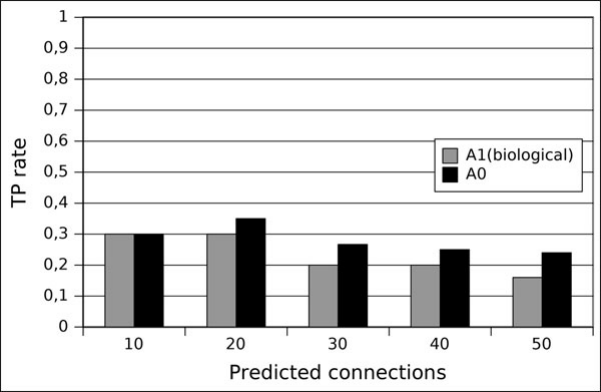
**True positive (HeLa)** Validation of the algorithm on the HeLa cells data. Plot of the true positive rate of predicted gene interactions computed from the highest ranks on two initial conditions: no biological knowledge (*ρ*_0,*i*_ -indicated as *A*_0_ in the label) and using biological knowledge (*ρ*_1,*i*_ - indicated as *A*_1_).

In Figure 8, we plot the true positive rate of the predicted interactions using the rank of undirected interactions. With no biological considerations (*ρ*_0,*i*_), the true positive rate stands between 17% and 30%.By adding some knowledge (*ρ*_1,*i*_), the true positive increases and stands between 25% and 35%.

#### Inducing a connection in HeLa cells data

**Figure 9:**
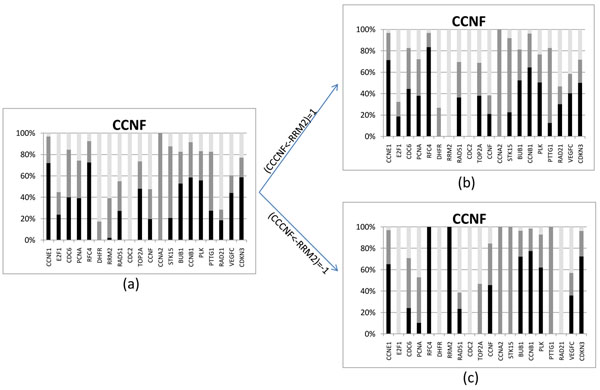
**Results of a connection induction of gene** Results of a connection induction of gene CCNF1 that most determine others. (a) Bar chart of initial frequencies. (b) Bar chart after connection CCNF←RRM2 determined as an activation. (c) Bar chart after connection CCNF←RRM2 determined as an inhibition.

To illustrate the method of inducing a connection which most determine others, we arbitrarily chose gene CCNF in the HeLa cells data. Executing the steps necessary to this analysis, we found that if the connection CCNF← RRM2 was determined as an inhibition the connections on CCNF from genes E2F1,RFC4, DHFR, STK15, PTTG1, and RAD21, would be determined as well (Figure 9).

The second highest rank produced is the connection CCNF← RAD21 determined as no relationship. As these two genes are classified as members of the same cell cycle phase, and the experimental determination of “no relationship” between two genes is difficult, we do not consider this result relevant.

### Discussion

By looking at Figures 2, 3, 6 and 7, it is interesting to note that, in some cases, the frequency analysis of connections was capable of almost excluding one relationship possibility, transforming some undetermined connections into partially determined connections. These results show that the cell cycle pathway constrains some connections, therefore restricting the whole network [34].

We can attribute this phenomenon to the high dependency that the determination of a gene connection has on other connections. The proposed algorithm performs a search over the space of possibilities of the influence of a set of genes over a single gene. If one of these influences is *a priori* determined (or known), this result can bias other connections. For example, let us suppose that genes A and B have to produce a positive output over a gene C, according to some restriction imposed by the time-series data. If we already know that gene A has no relationship to gene C, gene B must have a positive relationship on gene C. Therefore, this high dependency on the determination of a gene connection over the network makes the use of Figures 2, 3, 6 and 7 very restricted. If we simply use a relationship with a high weight to be our “best guess” on the connection between two genes, this choice can constrain other relationships, leading the system to a more or less determined state, or even creating a connection in a network that is not consistent with the data.

Another fact to be pointed out is the importance of the inferred partially determined connections. Although these connections can not be directly used to construct a network like the determined connections, it can guide some biological experiments, since a partially determined connection states that at least one type of relationship between two genes is not possible.

We could use the connection frequencies generated to attribute a strength of connection to the relationship of a partially determined connection, e.g., in the yeast cell cycle, the interference of Clb1 on SBF can be stated as 80% (or a probability of 0.8) of being an inhibition. In fact, we use the frequencies in Figures 2, 3, 6 and 7 to compute the rank of undirected relationships (Equation 11).

Regarding the validation, in the yeast cell cycle data, which is artificially generated, the true positive rate (Figure 5) of predicted connections is very high (75%) when 25 connections are considered, and 100% for 5 and 10 connections. These results show that the proposed algorithm can be successfully applied in an artificial data, i.e., a data set without noise and good balance between time points and number of genes. Evidently, our rank procedure is constructed in a way that determined connections and partially determined connections would be benefited. Hence, as our algorithm correctly determines 13 directed connections in the yeast cell cycle model, its true positive rate for a small number of undirected predictions is high as well.

Considering the HeLa cells data, the results are not quite optimistic. The true positive rate (Figure 8) stands between 17% and 30% for the inference procedure without biological knowledge and 25% to 35% using biological knowledge. However, if we consider the small amount of time points (12 per cell cycle) and the number of genes (20 in this simplified version), the difficulty of obtaining a higher true positive rate is clear.

Evidently, the method here proposed can only be used to aid a wet lab experiment on finding gene interactions if considerations about the network size and amount of time-series data were made. In situations where a large set of *g* genes is investigated and only a small amount of time-series data is available, as in the HeLa cells data, we would recommend that a rank of the *r* first interactions, with *g* ≪ *r* <*g*^2^/2, to reduce the set of possible gene interactions to be tested.

To the HeLa cells, we can also explain the low true positive rate by considering the 20 genes version of the network too simplified. Maybe our algorithm predicts interactions that are not directly observed in nature, but only through a series of interactions of genes not present in our network. Therefore, our validation procedure is compromised.

It is worth to notice that adding some biological knowledge the results are improved for the HeLa cells. This fact reinforces the need for an integrated work with biologists in a network inference process, as we show that even using little pieces of biological information we can improve the whole procedure.

Regarding the example of finding a connection which most determines others, we expect to exemplify here that this use of the algorithm could substantially aid biological experiments. It is also worth noticing that the connection found, CCNF← RRM2 as an inhibition, could make biological sense, as RRM2 is classified as a gene of the S phase and CCNF is a G_2_ phase gene. As the connection CCNF← RRM2, defined as an activation, is also well ranked, we can say that this relation is worth for an empirical test.

A closer look at the frequency analysis raises another interesting question: would not the network chosen by nature be easily detectable? Or even better: would not the utilized data be enough to constrain the connection frequencies into nature’s choice? We could answer this question by pointing out a truth that unequivocally distinguishes our model from nature’s choice: the chemical interactions between proteins. Evidently, some of the connections considered on many steps of the algorithm here presented cannot exist due to chemical incompatibilities. In some sense, nature has more information to constrain its network than we do.

## Conclusions

This paper proposes an algorithm to perform analyses for discovering gene regulatory interactions from time-series data under the Boolean network model and in the context of Constraint Satisfaction Problem (CSP). In fact, the inference of gene regulatory network is a one-to-many inverse problem in the sense that there may exist several networks consistent with the dataset. In order to analyze the gene interactions, we have generated several gene connections in consistent networks by using CSP solver techniques which in turn utilized constraints sets built from three algorithms provided by this work. We have applied our methodology to an artificial dataset that had been generated by a Boolean network that models the budding yeast cell cycle [35], and to an experimental dataset of HeLa cells [36]. By these applications, we have shown that our analyses could be a first step for detection of gene relationships with a high flexibility to include biological knowledge.

A challenge always presented in any gene regulatory model is its usefulness. It would be very interesting if a model could help biological experiments in understanding gene interactions. The model presented here together with the algorithm proposed is a first step to aid an inference process from time-series data of gene expression, and it can be improved by all a *priori* knowledge available. As it was made clear in the HeLa cells data, the use of biological knowledge can improve the efficiency of the proposed algorithm. For future steps, an interesting feature to be improved on our method is the ability to indicate which connection should be verified in the wet lab to help determine others. As exemplified in this work, this feature could lead to important contributions on wet lab experiments. To use this method in an empirical gene connection survey, we would recommend a search over all possible connections between all genes, and then proceed with the ranking process. Evidently, biological considerations over the highest ranks produced is heavily necessary.

However, there are other characteristics to be sought that could constrain the network towards nature’s choice. On one hand, so far, only constraints built from successive states are considered. Thus, constraints constructed from considering the whole trajectory (e.g., some kind of powers of the regulation matrix in order consider succession of more than two or three states) could help to obtain more precise solutions. In fact, although we have carefully generated uniform samples to build a set of solutions (to produce representations good enough for connection frequencies) by using an appropriate heuristic for variable and value ordering, it is important to keep in mind that in order to make a more precise frequency analysis, one needs the *consistent solutions* in the CSP context, meaning that, in our case, the solutions obtained from considering the whole trajectory. On the other hand, one feature not explored in this paper is the dynamics of the network. There are indications, as stated by Kauffman [23], that nature would prefer networks with a small amount of attractors - the gene pattern expression that leads the system to itself -and large basins of attraction - the set of gene pattern expressions that leads the system to an attractor. The network assembled by Li et al. [35] has these characteristics. Therefore, an analysis of connections computed only from networks with a few number of attractors - or other dynamical characteristic - could create a well established result. One naïve way to proceed is to build regulation matrices from the solutions of the CSPs subproblems (possible rows) and select the ones such that present the dynamical features described before (large basins of attraction and small number of attractors)

Concluding, the analysis presented here is a remarkable first step for the construction of a system to infer gene interactions. The true positive rate on the artificial data is excellent and, considering noises and lack of time points, the true positive rate for the experimental data is beyond expectation.

We understand that any inference procedure can not have success if it does not contain biological and computational expertise, therefore the future steps of this research tend to be centered on the difficulties of a wet lab, or its limitations.

## Competing interests

The authors declare that they have no competing interests.

## Authors’ contributions

RFH headed the research. All participated in the manuscript development. All authors read and approved the final manuscript. Carlos H A Higa and Vitor H P Louzada have contributed equally to this work.

## Supplementary Material

Additional file 1 **Bar charts for *A*_0_** The bar charts for the 20 genes are available at: http://yeast.ime.usp.br/hela/additional_files1.zip The additional files1.zip (235.5 KB) contains charts in PDF format. Click here for file

Additional file 2 **Bar charts for *A*_1_** The bar charts for the 20 genes are available at: http://yeast.ime.usp.br/hela/additional_files2.zip The additional files2.zip (234.4 KB) contains charts in PDF format. Click here for file
