# Supplementary material for: Constraint-based analysis of gene interactions using restricted boolean networks and time-series data
Source: BMC Proc. 2011 May 28;5(Suppl 2):S5. doi: 10.1186/1753-6561-5-S2-S5 (PMC3090763; doi:10.1186/1753-6561-5-S2-S5)
Supplement: Additional file 1 — Bar charts for A0 The bar charts for the 20 genes are available at: http://yeast.ime.usp.br/hela/additional_files1.zip The additional files1.zip (235.5 KB) contains charts in PDF format. [file 1753-6561-5-S2-S5-S1.pdf]

Additional file 1.

Bar charts for *A*0

The bar charts for the 20 genes are available at:
http://yeast.ime.usp.br/hela/additional_files1.zip

The additional files1.zip (235.5 KB) contains charts in PDF format.
