# Supplementary material for: Constraint-based analysis of gene interactions using restricted boolean networks and time-series data
Source: BMC Proc. 2011 May 28;5(Suppl 2):S5. doi: 10.1186/1753-6561-5-S2-S5 (PMC3090763; doi:10.1186/1753-6561-5-S2-S5)
Supplement: Additional file 2 — Bar charts for A1 The bar charts for the 20 genes are available at: http://yeast.ime.usp.br/hela/additional_files2.zip The additional files2.zip (234.4 KB) contains charts in PDF format. [file 1753-6561-5-S2-S5-S2.pdf]

Additional file 2.

Bar charts for *A*1

The bar charts for the 20 genes are available at:
http://yeast.ime.usp.br/hela/additional_files2.zip

The additional files2.zip (234.4 KB) contains charts in PDF format.
